# *Bacteroides celer* sp. nov. and *Bacteroides mucinivorans* sp. nov., isolated from human feces, and the reclassification of *Bacteroides koreensis* Shin et al. 2017 and *Bacteroides kribbi* Shin et al. 2017 as later heterotypic synonyms of *Bacteroides ovatus* Eggerth and Gagnon 1933 (Approved Lists 1980)

**DOI:** 10.71150/jm.2502006

**Published:** 2025-06-30

**Authors:** Ah-In Yang, Bora Kim, Woorim Kang, Hae-In Joe, Na-Ri Shin

**Affiliations:** 1 Biological Resource Center, Korea Research Institute of Bioscience and Biotechnology, Jeongeup 56212, Republic of Korea; 2 Department of Biology, Kyung Hee University, Seoul 02447, Republic of Korea; 3 Department of Biological Science, Kangwon National University, Chuncheon 24341, Republic of Korea

**Keywords:** *Bacteroides celer*, *Bacteroides mucinivorans*, *Bacteroides ovatus*, human gut microbiota, reclassification

## Abstract

Two novel, Gram-stain-negative, anaerobic, and non-motile bacterial strains, designated KFT8^T^ and CG01^T^, were isolated from the feces of healthy individuals without diagnosed diseases and characterized using a polyphasic approach. Phylogenetic analysis revealed that both strains belong to the genus *Bacteroides*, with < 99.0% similarity in their 16S rRNA gene sequences to *B. facilis* NSJ-77^T^ and *B. nordii* JCM 12987^T^. Within the genus *Bacteroides*, strain KFT8^T^ exhibited the highest Orthologous Average Nucleotide Identity value of 94.7% and a digital DNA-DNA hybridization value of 63.7% with *B. ovatus* ATCC 8483^T^, whereas strain CG01^T^ showed the highest values of 95.3% and 63.3%, respectively, with *B. nordii* JCM 12987^T^. The values between the two novel strains were 74.8% and 21.4%, respectively, which are below the species delineation thresholds, supporting their classification as novel species. The major fatty acid of strain KFT8^T^ was C_18:1_ *ω*9*c*, whereas strain CG01^T^ predominantly contained summed feature 11 (comprising *iso*-C_17:0_ 3OH and/or C_18:2_ DMA). The only respiratory quinone was MK-11, the major polar lipid was phosphatidylethanolamine. Both strains produced succinic acid and acetic acid as common metabolic end-products of fermentation, while lactic acid and formic acid were detected individually in each strain. Based on polyphasic characterization, strains KFT8^T^ (= KCTC 15614^T^ = JCM 36011^T^) and CG01^T^ (= KCTC 15613^T^ = JCM 36010^T^) represent two novel species within the genus *Bacteroides*, for which the names *Bacteroides celer* sp. nov. and *Bacteroides mucinivorans* sp. nov. are proposed, respectively. Additionally, genome-based analyses and phenotypic comparisons revealed that *B. koreensis* and *B. kribbi* represent the same strain, showing genomic relatedness to *B. ovatus* that exceeds the threshold for species delineation. Consequently, we propose the reclassification of *B. koreensis* Shin et al. 2017 and *B. kribbi* Shin et al. 2017 as later heterotypic synonyms of *B. ovatus* Eggerth and Gagnon 1933 (Approved Lists 1980).

## Introduction

The gut microbiota is known to play a crucial role in host health and disease development. The 10^13^–10^14^ bacteria of the human gut microbial community significantly impact human health and physiology (Wardman et al., 2022). These microorganisms actively engage in both positive and negative interactions with their surrounding environment, such as the gastrointestinal (GI) mucus layer, metabolites, and other microbes in the community. As a result, they contribute significantly to various aspects, including nutrient acquisition (Martens et al., 2014), immune system development (Kau et al., 2011), drug metabolism (Zimmermann et al., 2019), and formation of a complex microbial community through cross-feeding interactions (Culp and Goodman, 2023). The GI mucus layer is composed of various components forming a complex that includes water, electrolytes, lipids, and specific mucus proteins known as mucin, which serves as the initial line of defence in the GI tract, acting as a crucial physical barrier against infectious diseases caused by invasive pathogens (Desai et al., 2016).

The genus *Bacteroides* is one of the predominant genera in human GI tracts (Mahowald et al., 2009) and plays a key role in the degradation of polysaccharides. As of February 2025, the genus *Bacteroides* includes 78 validly published species (excluding synonyms) out of 161 total species. This count includes species with preferred names that are not validly published (https://lpsn.dsmz.de/genus/bacteroides) (Parte et al., 2020). The genus *Bacteroides* was introduced by Castellani and Chalmers in 1919, with *Bacteroides fragilis* as the type species (Castellani and Chalmers, 1919). However, its earlier designation was the basonym *Bacillus fragilis*, proposed by Veillon and Zuber in 1898 (Veillon and Zuber, 1898). *B. fragilis* was officially designated as the type species of the genus *Bacteroides* in 1980. Species of this genus are Gram-stain-negative, strictly anaerobic, rod-shaped bacteria, with *anteiso*-C_15:0_ as the predominant cellular fatty acid (Chassard et al., 2008; Song et al., 2004; Wexler, 2007). To investigate the mucin-associated bacterial community, we conducted a culture-based polyphasic study. During this study, we isolated two novel bacterial species, designated strains KFT8^T^ and CG01^T^, which are closely related to the genus *Bacteroides*, from human feces inoculated into a mucin-minimal medium.

A previous study proposed *B. koreensis* YS-aM39^T^ and *B. kribbi* R2F3-3-3^T^ as two novel species within the genus *Bacteroides*, exhibiting the highest 16S rRNA gene sequence similarities to *B. ovatus* (Shin et al., 2017). However, during our comparative analysis of a newly isolated human gut bacterium, strain KFT8^T^, it was observed that the genomic and biochemical characteristics of the two species, *B. koreensis* and *B. kribbi*, could not be distinguished from each other or from those of *B. ovatus* at the species level. In this study, we report that strains KFT8^T^ and CG01^T^ represent two novel species within the genus *Bacteroides* based on taxonomic characterization. Additionally, we propose the reclassification of *B. koreensis* and *B. kribbi* as later heterotypic synonyms of *B. ovatus*.

## Materials and Methods

### Isolation and deposition of bacterial strains

To investigate the mucin-associated bacterial community, fresh fecal samples were collected from non-hospitalized, disease-free individuals (hereafter referred to as healthy) in Korea and immediately transferred to an anaerobic chamber (Bactron II-2, Sheldon Manufacturing Inc.) filled with a gas mixture of 5% H_2_, 5% CO_2_, and 90% N_2_. Subsequently, 1 g of the fecal sample was inoculated into 25 ml of mucin-minimal medium and incubated at 37°C. The mucin-minimal medium was prepared as described by Shin et al. (2018). All media and solutions were pre-reduced at least 2 days before use in the anaerobic chamber. After 2 weeks of incubation, the inoculum was diluted to a 10^-7^ dilution using phosphate-buffered saline, which had been adjusted to a pH of 7.4 with HCl (Bioneer), and the resulting dilutions were spread on tryptic soy agar (TSA; BD) containing 0.3% (w/v) yeast extract and 10% (v/v) rumen fluid (Bar Diamond Inc.), brain heart infusion (BHI; BD) agar plates with 0.25% (w/v) porcine gastric mucin-type III (Sigma-Aldrich), and Gifu Anaerobic Medium (GAM; Nissui Pharmaceutical) agar plates with 0.1% (v/v) vitamin K1-hemin solution (BD BBL).

During the colony identification process using 16S rRNA gene sequence analysis, two *Bacteroides*-like bacterial strains, designated KFT8^T^ and CG01^T^ (hereafter referred to as two novel strains), were isolated. Strain KFT8^T^ was isolated from a TSA plate supplemented with yeast extract and rumen fluid, inoculated with a fecal sample from a 23-year-old healthy male. Strain CG01^T^ was isolated from a GAM plate supplemented with a vitamin K1-hemin solution, inoculated with a fecal sample from a 28-year-old healthy female. The two novel strains were maintained on the GAM agar plates and repeatedly transferred to obtain pure cultures. The purified isolates were preserved in GAM broth containing 40% glycerol at -80°C for cryopreservation. The two novel strains were deposited at the Korean Collection for Type Cultures (KCTC) and the Japan Collection of Microorganisms (JCM). The accession numbers are KCTC 15614^T^ and JCM 36011^T^ for strain KFT8^T^, and KCTC 15613^T^ and JCM 36010^T^ for strain CG01^T^.

### 16S rRNA gene phylogeny

In the colony PCR of the two novel strains, the 16S rRNA genes were amplified using bacterial universal primers 27F (5՛-AGAGTTTGATCCTGGCTCAG-3՛) and 1492R (5՛-TACGGYTACCTTGTTACGACTT-3՛) with a PCR pre-mix (iNtRON Biotechnology Inc.) using a C1000 Touch^TM^ Thermal Cycler (Bio-Rad) (Lane, 1991). PCR products were sequenced, and reads were assembled as described by Yang et al. (2024). The 16S rRNA gene sequences of the two novel strains were compared to those of validly published type strains using the EzBioCloud server (https://www.ezbiocloud.net/) (Yoon et al., 2017) and aligned using CLUSTAL W implemented in BioEdit (Hall, 1999).

The pairwise nucleotide sequence alignment tool on the EzBioCloud server was used to compare the two novel strains and assess their sequence similarity and difference. Phylogenetic consensus trees were then constructed using three algorithms: neighbor-joining (NJ) (Saitou and Nei, 1987), maximum-parsimony (MP) (Fitch, 1971), and maximum-likelihood (ML) (Felsenstein, 1981), with 1,000 bootstrap replications in MEGA 11 (Felsenstein, 1985; Tamura et al., 2021). The evolutionary distances were computed using the Tamura-Nei model (Tamura et al., 2004). *Parabacteroides distasonis* JCM 5825^T^ (NR041342) was selected as an outgroup for the phylogenetic trees.

During the phylogenetic analysis, we found erroneous mismatches in the conserved regions of the 16S rRNA gene sequences of *B. koreensis* KCTC 15520^T^ (GenBank accession number KX025133) and *B. kribbi* KCTC 15460^T^ (GenBank accession number KX025134). To correct these discrepancies, revised 16S rRNA gene sequences of *B. koreensis* KCTC 15520^T^ and *B. kribbi* KCTC 15460^T^ were obtained using the same method.

### Genomic analyses

The genomic DNA of the two novel strains was extracted using the MG^TM^ Genomic DNA Purification Kit (Macrogen, Korea) following the manufacturer’s protocol. Briefly, bacterial cells were lysed with lysozyme, treated with RNase, purified using AL and PP buffers, and precipitated using isopropanol. The DNA pellet was washed with ethanol, dried, and then resuspended in distilled water. For sequencing, DNA libraries were prepared using the SMRTbell Express Template Prep Kit 2.0 (PacBio) and the TruSeq Nano DNA High Throughput Library Prep Kit (Illumina). Illumina libraries were quantified using quantitative PCR (qPCR) with the KAPA Library Quantification Kit and assessed using the Agilent 4200 TapeStation D1000 ScreenTape. Whole-genome sequencing was performed using a hybrid approach, combining PacBio long-read sequencing and Illumina Miseq short-read sequencing. De novo assembly of PacBio reads was performed using CANU v2.2, with Illumina reads incorporated for error correction. The resulting draft genomes were polished with Pilon v1.22 to correct misassemblies, fix base errors, and fill gaps.The average nucleotide identity (ANI) values, calculated using the OrthoANI algorithm, and digital DNA-DNA hybridization (dDDH) values were used to assess the genomic relatedness between each of the two novel strains and the most closely related species. These evaluations were conducted using genome-to-genome calculator v3.0 on the DSMZ server (http://ggdc.dsmz.de/ggdc.php) (Meier-Kolthoff et al., 2013) and Orthologous Average Nucleotide Identity Tool (OAT) (Lee et al., 2016), respectively. The phylogenomic tree of the two novel strains, along with phylogenetically related species, was constructed using an up-to-date bacterial core genes 2 (UBCG 2) pipeline (Kim et al., 2021) and the FastTree program (Price et al., 2009). Whole genomes of closely related type species were obtained from GenBank.

Functional annotation of protein-coding sequences (CDSs) was performed using the Rapid Annotation using Subsystem Technology (RAST) server (https://rast.nmpdr.org/) (Aziz et al., 2008). To investigate putative differences in carbohydrate utilization between the two novel strains, we employed the dbCAN3 meta server pipeline for automated Carbohydrate-Active Enzymes (CAZymes) annotation (https://bcb.unl.edu/dbCAN2/index.php) (Zheng et al., 2023). Three different annotation tools (HMMER: dbCAN, DIAMOND: CAZy, and HMMER: dbCAN-sub) were utilized for accurate prediction and annotation of CAZymes in their genomes (Buchfink et al., 2015; Finn et al., 2011). To improve precision, hits not identified by at least two out of three tools were excluded from further analysis.

To ascertain the potential of *B. koreensis* JCM 31393^T^ (GenBank accession number VJZV00000000) and *B. kribbi* JCM 31391^T^ (GenBank accession number VJYL00000000) as heterotypic synonyms of *B. ovatus* ATCC 8483^T^ (GenBank accession number CP012938), we evaluated genomic distances among the three strains using the method described earlier in this section.

### Morphological, physiological, and biochemical features

For comparative analyses, validly published type strains were selected based on 16S rRNA gene sequence similarity and phylogenetic relatedness. For strain KFT8ᵀ, the selected strains included *B. facilis* KCTC 25155ᵀ, *B. ovatus* KCTC 5827ᵀ, and *B. xylanisolvens* KCTC 15192ᵀ. For strain CG01ᵀ, *B. nordii* KCTC 25023ᵀ and *B. salyersiae* KCTC 5799ᵀ were selected. All type strains were obtained from KCTC. To assess morphological features, the two novel strains and the closely related species were cultivated on GAM agar at 37°C for 3 days. Cell morphology of the two novel strains was observed using an energy-filtering transmission electron microscope (LIBRA 120, Carl Zeiss) and a light microscope (Eclipse 50*i*, Nikon). Gram reaction was determined by observing the string reaction using a 3% (w/v) potassium hydroxide solution (Halebian et al., 1981). Endospore formation was determined by spore staining using malachite green and safranin solutions (Sigma-Aldrich) in strains incubated on GAM agar at 37°C for 7 days (Schaeffer and Fulton, 1933). Aerobic growth was determined on GAM agar at 37°C in the ambient atmosphere. Oxidase activity was verified by observing a color change to purple in the colonies, indicating indophenol blue production after adding 1% (w/v) tetramethyl-p-phenylenediamine (bioMérieux). Catalase activity was confirmed by observing bubble production upon the addition of 3% (v/v) hydrogen peroxide. Motility was investigated using 0.5% semi-solid GAM agar (Tittsler and Sandholzer, 1936). Bile tolerance was determined by cultivation on GAM agar containing 2% oxgall (dehydrated bile; BD). The physiological features of the two novel strains were determined by performing a growth test in GAM broth. Growth range and optimal growth conditions were determined at 10, 20, 25, 30, 37, 40, 42, and 55°C. The pH range and optimal pH conditions were assessed using media adjusted to pH values ranging from 4 to 11 at intervals of 1. The pH was adjusted using 6 N HCl or 10 N NaOH, with the following buffers applied: 2-morpholino-ethanesulfonic acid (MES) at pH 4–6, 4-(2-hydroxyethyl)piperazine-1-ethanesulfonic acid (HEPES) at pH 7–9, and 3-(cyclohexylamino)-2-hydroxy-1-propanesulfonic acid (CAPSO) at pH 10–11. NaCl tolerance was evaluated using GAM broth supplemented with NaCl to final concentrations of 0, 1, 2, 3, 4, 5, 7, 10, 12, 15, and 20% (w/v), with the 0% condition prepared by diluting the broth to 0.3× to minimize basal NaCl content. Growth rates were assessed by measuring the optical density of the cultures at 600 nm (OD_600_) after 24 h, 48 h, and 7 days of incubation using a spectrophotometer. Biochemical features, including the ability to produce acids from carbon sources, the utilization of carbon sources as sole growth substrates, and enzyme profiles, were assessed and compared with their closely related type strains. For this assessment, we utilized API 50CH test strips with 50CHB/E medium, API Rapid ID 32A, and API 20A test strips (bioMérieux), following the manufacturer’s instructions, and bacterial cultures grown on GAM agar at 37°C for 3 days. Fermentation end-product profiling was determined by analyzing the supernatant from bacterial cultures using an HPLC system (Shimadzu) equipped with Aminex^TM^ Organic Acid Columns (Bio-Rad). Cell-free cultures were obtained by centrifugation (7,500 × g, 30 min, and 4°C) and filtration (0.22 μm pore size) from cultures grown on the GAM broth at 37°C for 3 days. After injection, the column was isocratically eluted with 5 mM sulfuric acid solution at a flow rate of 0.7 ml/min for 25 min.

### Chemotaxonomy

The cellular fatty acids from the two novel strains and the closely related species, cultured on GAM agar at 37°C for 3 days, were extracted using the protocol described in the Sherlock® Microbial Identification System (MIDI) (Sasser, 1990). The analysis and identification of cellular fatty acid profiles were performed using a gas chromatograph (Agilent 6890; Agilent Technologies) with the MIDI software package (Sherlock version 6.2), based on the Moore library database v6.00. Polar lipids and respiratory quinones from the two novel strains were extracted using a chloroform:methanol (2:1, v/v) solvent mixture from freeze-dried cells obtained after cultivation on GAM agar at 37°C for 3 days (Collins and Jones, 1981). Polar lipid analysis was performed using two-dimensional thin-layer chromatography (TLC) on silica gel plates (Merck) (Goodfellow et al., 1976). The first-dimensional development used chloroform:methanol:distilled water (65:25:4, v/v/v), and the second-dimensional development used chloroform:methanol:acetic acid:distilled water (80:15:12:4, v/v/v/v). Polar lipid spots on the TLC plates were detected using four spray reagents: molybdatophosphoric acid for total lipids, ninhydrin reagent for amino group-containing lipids, Zinzadze reagent for phospholipids, and *α*-naphthol reagent for glycolipids. The extracted quinones were purified by development on silica gel TLC plates (Merck) using a solvent system of petroleum ether:diethyl ether (85:15, v/v). Respiratory quinones were analyzed using a Shimadzu HPLC system (Shimadzu Corporation) comprising LC-20AD pumps, a CTO-20A column oven, a CBM-20A communication module, a DGU-20A degassing unit, and an SPD-20A UV/VIS detector. Separation of the quinones was performed isocratically on a ZORBAX SB-C18 ODS column (Agilent Technologies) using methanol:diisopropyl ether (3:1, v/v) as the mobile phase, and detection was carried out at a UV wavelength of 270 nm. All physiological, chemotaxonomic, and biochemical analyses were conducted in at least three replicates.

### Accession numbers

The GenBank/EMBL/DDBJ accession numbers for the 16S rRNA gene and whole-genome sequences of strain KFT8^T^ are KY703634 and CP178209, respectively, while those for strain CG01^T^ are OR272335 and CP178179. The revised 16S rRNA gene sequences of *B. koreensis* KCTC 15520^T^ and *B. kribbi* KCTC 15460^T^ have been deposited under OR226560 and OR226559.

## Results and Discussion

### 16S rRNA gene phylogeny

The pairwise 16S rRNA gene sequence similarity between strains KFT8^T^ and CG01^T^ was 95.2%, with 69 nucleotide differences, suggesting that the two novel strains can be differentiated based on their 16S rRNA gene sequences. Strain KFT8^T^ exhibited high 16S rRNA gene sequence similarity with *Bacteroides facilis* NSJ-77^T^, *B. ovatus* ATCC 8483^T^, *B. koreensis* YS-aM39^T^, and *B. xylanisolvens* XB1A^T^ (98.9%, 98.8%, 97.8%, and 97.4%, respectively). Strain CG01^T^ showed 99.0% and 96.9% similarity to *B. nordii* JCM 12987^T^ and to *B. salyersiae* WAL10018^T^, respectively.

**Fig. 1.**
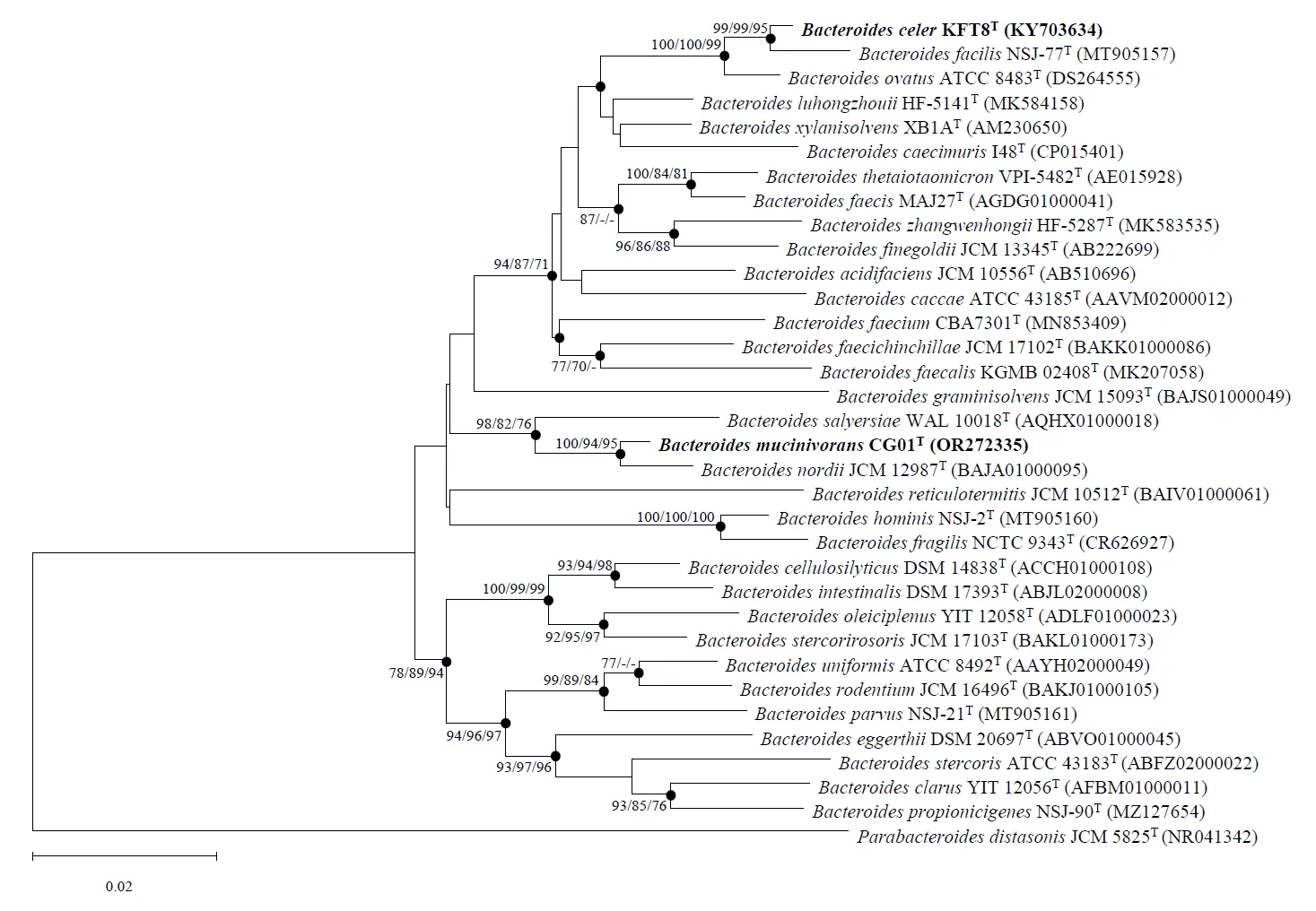
Phylogenetic tree based on 16S rRNA gene sequences constructed using NJ algorithm. The phylogenetic positions of the two novel strains are shown relative to related species. Bootstrap values over 70% from NJ, ML, MP algorithms are indicated at nodes as percentages from 1,000 replicates. Filled circles indicate branches consistently recovered by all three algorithms. The scale bar indicates 0.02 substitutions per nucleotide.

Strains KFT8^T^ and CG01^T^ each clustered with type strains exhibiting high 16S rRNA gene sequence similarity (Fig. 1). Strain KFT8^T^ clustered with *B. facilis* NSJ-77^T^, *B. ovatus* ATCC 8483^T^, and *B. xylanisolvens* XB1A^T^, supported by high bootstrap values (100/100/99 for NJ/ML/MP). Within this group, strain KFT8^T^ formed a monophyletic clade with *B. facilis* NSJ-77^T^ and *B. ovatus* ATCC 8483^T^, showing a sister-taxon relationship with *B. facilis* NSJ-77^T^. Although *B. xylanisolvens* XB1A^T^ exhibited the third-highest 16S rRNA gene sequence similarity (97.4%), it diverged from the clade, indicating that strain KFT8^T^ is more closely related to *B. facilis* NSJ-77^T^ and *B. ovatus* ATCC 8483^T^ than to *B. xylanisolvens* XB1A^T^. Strain CG01^T^ formed a monophyletic clade with *B. nordii* JCM 12987^T^ and *B. salyersiae* WAL 10018^T^. Within this clade, strain CG01^T^ exhibited the closest phylogenetic relationship with *B. nordii* JCM 12987^T^, supported by high bootstrap values (100/94/95). *B. salyersiae* WAL 10018^T^ also showed a close relationship with strain CG01^T^, as indicated by high bootstrap values (98/82/76) and 16S rRNA gene sequence similarity (96.9%), suggesting significant phylogenetic relatedness. Based on phylogenetic analyses, *B. facilis* KCTC 25155^T^, *B. ovatus* KCTC 5827^T^, and *B. xylanisolvens* KCTC 15192^T^ were employed to compare phenotypic, genomic, and chemotaxonomic properties with strain KFT8^T^, while *B. nordii* KCTC 25023^T^ and *B. salyersiae* KCTC 5799^T^ were used for comparative analysis with strain CG01^T^.

The revised sequences yielded almost full-length sequences of 1,465 bp for *B. koreensis* KCTC 15520^T^ (GenBank accession number OR226560) and 1,463 bp for *B. kribbi* KCTC 15460^T^ (GenBank accession number OR226559). Pairwise comparisons of the revised 16S rRNA gene sequences revealed 100% similarity between *B. koreensis* KCTC 15520^T^ and *B. kribbi* KCTC 15460^T^, with both sharing 99.5% sequence similarity to *B. ovatus* ATCC 8483^T^. *B. ovatus* ATCC 8483^T^, *B. koreensis* KCTC 15520^T^, and *B. kribbi* KCTC 15460^T^ formed a well-supported monophyletic clade with high bootstrap values (100/98/99). However, the branch length between *B. koreensis* KCTC 15520^T^ and *B. kribbi* KCTC 15460^T^ was nearly zero, indicating minimal genetic divergence. Although both species diverged from *B. ovatus* ATCC 8483^T^ at an earlier branching point, all three strains exhibited very high 16S rRNA gene sequence similarity (99.5–100%). This topology was consistent across all three algorithms (Fig. S1, available in the online version of this article). These findings suggest that *B. koreensis* and *B. kribbi* may not represent distinct species but rather strains of *B. ovatus*. Although 16S rRNA gene-based analyses indicate close relatedness among the three species, these results lack the resolution required for definitive species delineation. Therefore, we further evaluated their genomic relatedness using whole-genome analyses to determine whether taxonomic reclassification is warranted.

### Genomic analyses

**Fig. 2.**
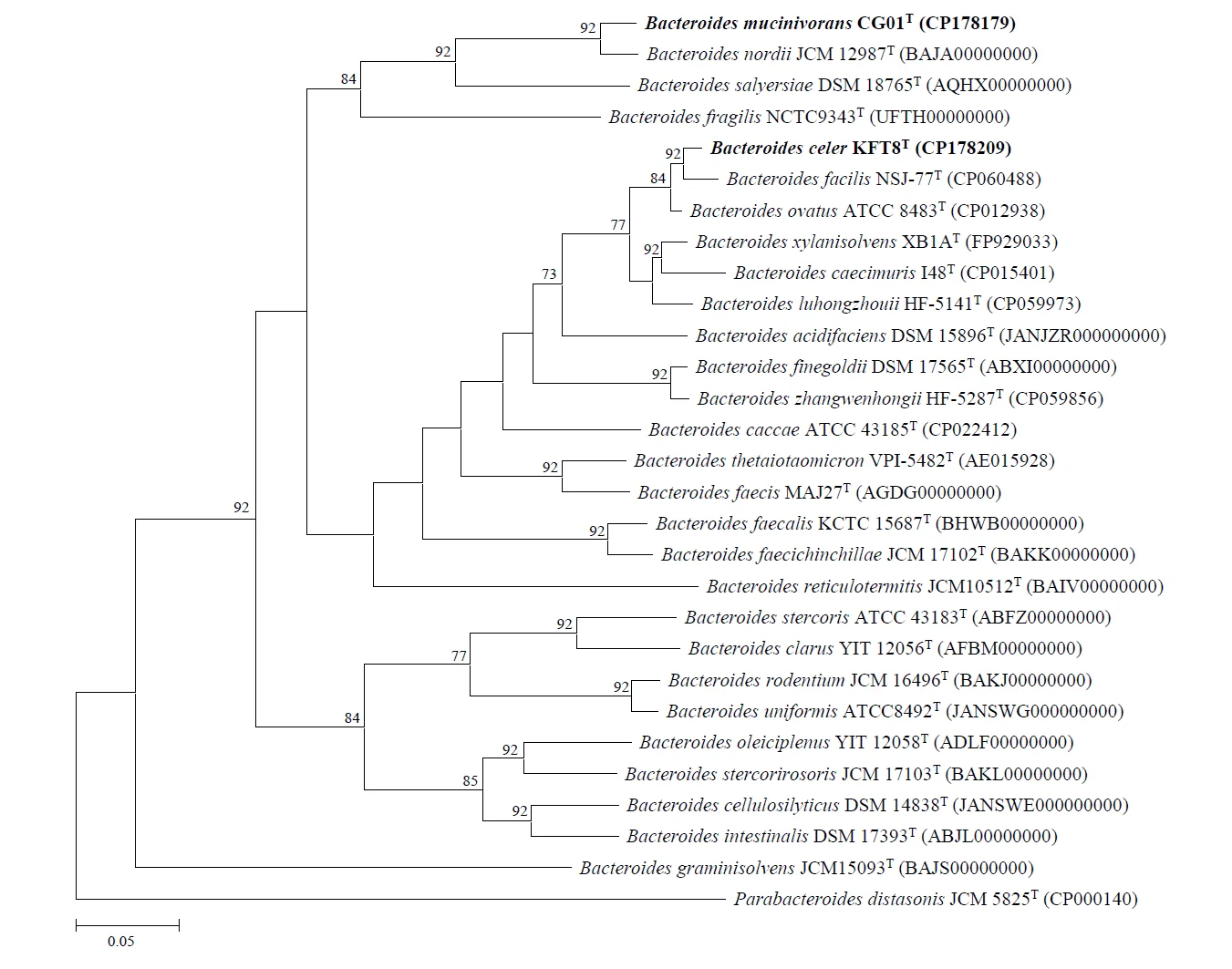
Phylogenomic tree of the two novel strains and closely related species based on genome sequences, constructed using the UBCG pipeline. Numbers at nodes represent bootstrap values. The scale bar indicates 0.05 substitutions per nucleotide.

Genome sequencing of strains KFT8^T^ and CG01^T^ yielded single complete contigs with coverages of 44.9× and 49×, respectively. The genome of strain KFT8^T^ was 6,031,385 bp in length, with a G + C content of 41.8%, and contained 4,910 predicted CDSs, 15 rRNA genes, and 24 tRNA genes. The genome of strain CG01^T^ was 6,053,331 bp in length, with a G + C content of 40.3%, and contained 4,727 CDSs, 15 rRNA genes, and 23 tRNA genes. A summary of the general genomic features of the two novel strains and their closely related species is provided in Table S1 (available in the online version of this article). The OrthoANI and dDDH values between strains KFT8^T^ and CG01^T^ were 74.8% and 21.4%, respectively (Table S2, available in the online version of this article). Strain KFT8^T^ showed the highest genomic relatedness with *B. ovatus* ATCC 8483^T^ (94.7% OrthoANI; 63.7% dDDH), *B. facilis* NSJ-77^T^ (94.5% OrthoANI; 61.8% dDDH), and *B. xylanisolvens* XB1A^T^ (91.1% OrthoANI; 45.7% dDDH) among validly published type species. Strain CG01^T^ exhibited the highest genomic similarity with *B. nordii* JCM 12987^T^ (95.3% OrthoANI; 63.3% dDDH) and *B. salyersiae* DSM 18765^T^ (80.0% OrthoANI; 23.8% dDDH). Although the OrthoANI value of strain CG01^T^ (95.3%) is within the species delineation threshold range (95–96%), its corresponding dDDH value (63.3%) is below the accepted threshold (70%), strongly supporting its status as a novel species. In contrast, both the OrthoANI and dDDH values for strain KFT8^T^ are below the threshold, further confirming its distinctiveness from phylogenetically related species within the *Bacteroides* genus (Goris et al., 2007; Riesco and Trujillo, 2024). The phylogenomic tree showed that strain KFT8^T^ formed a paraphyletic branch with phylogenetically related type strains, including *B. facilis* NSJ-77^T^, *B. ovatus* ATCC 8483^T^, and *B. xylanisolvens* XB1A^T^, whereas strain CG01^T^ formed a monophyletic branch with *B. nordii* JCM 12987^T^ and *B. salyersiae* DSM 18765^T^ (Fig. 2).

The RAST analysis showed that the predominant subsystem categories in the two novel strains were ‘carbohydrates’, ‘amino acids and derivatives’, ‘protein metabolism’, and ‘cofactors, vitamins, prosthetic groups, pigments’, with minor differences in the predicted CDS numbers (Table S3, available in the online version of this article). The two novel strains possessed a total of 487 and 474 CAZyme genes, respectively, including glycoside hydrolase (GH), glycosyl transferase (GT), carbohydrate-binding module (CBM), and carbohydrate esterase (CE). Among these, GH was the predominant CAZyme family in both strains, comprising more than 287 GH genes and accounting for over 60% of the total (Table S4, available in the online version of this article). Similar to other members of the genus *Bacteroides*, the two novel strains are expected to metabolize various types of fiber, including both dietary and host-derived fibers, converting them into oligo- or monosaccharides.

Similar to the analysis based on the 16S rRNA gene, the OrthoANI and dDDH values between the type strains of *B. koreensis* (GenBank accession number VJZV00000000) and *B. kribbi* (GenBank accession number VJYL00000000) were determined to be 99.9% and 100%, respectively, indicating that they represent the same strain. Additionally, *B. koreensis* JCM 31393^T^ and *B. kribbi* JCM 31391^T^ exhibited an OrthoANI value of 98.5% and dDDH values of 87.8–87.9% with *B. ovatus* ATCC 8483^T^ (GenBank accession number CP012938) (Table S5, available in the online version of this article). In the phylogenomic tree, *B. koreensis* JCM 31393ᵀ, *B. kribbi* JCM 31391ᵀ, and *B. ovatus* ATCC 8483ᵀ formed a well-supported monophyletic clade with extremely short branch lengths. No distinct phylogenetic separation was observed between *B. koreensis* JCM 31393ᵀ and *B. kribbi* JCM 31391ᵀ, and both strains clustered tightly with *B. ovatus* ATCC 8483ᵀ. This topology strongly supports the interpretation that *B. koreensis* and *B. kribbi* do not represent distinct species, but are genomically indistinguishable from *B. ovatus*, thereby warranting taxonomic reclassification (Fig. S2, available in the online version of this article). According to the proposed criteria for species delineation, the results of whole-genome sequence-based genomic relatedness and the phylogenomic analysis strongly indicate that *B. koreensis* and *B. kribbi* should be considered later heterotypic synonyms of *B. ovatus* (Goris et al., 2007; Richter and Rosselló-Móra, 2009). In accordance with the guidelines of the International Code of Nomenclature of Prokaryotes (ICNP), later heterotypic synonyms of validly published names lose their nomenclatural standing (Oren et al., 2023). Therefore, *B. koreensis* and *B. kribbi* should be treated as abandoned species and reclassified accordingly.

### Morphological, physiological, and biochemical features

Cells of the two novel strains were strictly anaerobic, Gram-stain-negative, oxidase- and catalase-negative, bile-tolerant, non-motile, non-spore-forming, and rod-shaped. Strain KFT8^T^ measured 2.0–3.0 μm in length and 1.0–1.5 μm in width, while strain CG01^T^ measured 2.0–3.5 μm in length and 1.0 μm in width (Fig. S3, available in the online version of this article). Colonies of both strains were circular to round, cream-colored, translucent, moist, and glistening, with entire margins. Genome annotation revealed only two sporulation-related genes and no motility-related genes in the two novel strains and their closely related species. These findings support the conclusion that the strains lack the genetic capacity for endospore formation and are non-motile, consistent with the characteristics of the genus *Bacteroides*. Strain KFT8^T^ exhibited growth at temperatures ranging from 25 to 37°C, while strain CG01^T^ grew at temperatures ranging from 20 to 42°C. Both strains showed optimal growth at 37°C. The two novel strains exhibited growth within a pH range of 6 to 9, with optimal growth at pH 8, and in NaCl concentrations of 0 to 5% (w/v), with optimal growth at 1%. Strains KFT8^T^ and CG01^T^ exhibited distinct enzyme activities compared to their closely related species, and these biochemical differences are summarized in Table 1. Notably, *B. koreensis* KCTC 15520^T^ and *B. kribbi* KCTC 15460^T^ showed identical results across all tested biochemical features. These results were consistent for strains obtained from JCM, further supporting the conclusion that *B. koreensis* and *B. kribbi* represent the same strain. A detailed comparison of enzyme activities and acid production among the two novel strains and their validly published reference species is provided in Table 1.

### Chemotaxonomy

Succinic acid was identified as the predominant fermentation end-product, comprising 62.1% and 44.7% of the total detected metabolites in strains KFT8ᵀ and CG01ᵀ, respectively. Both strains also produced minor amounts of acetic acid, with KFT8ᵀ additionally producing lactic acid and CG01ᵀ producing formic acid. Strain KFT8^T^ exhibited C_18:1_ *ω*9*c* as the predominant cellular fatty acid, accounting for 21% of the total. In contrast, strain CG01^T^ showed a predominance of summed feature 11, comprising *iso*-C_17:0_ 3OH and/or C_18:2_ DMA, which accounted for 27.2% of the total. The cellular fatty acid profiles of the two novel strains and their closely related species are summarized in Table 2. Menaquinone-11 (MK-11) was identified as the sole respiratory quinone in the two novel strains. Polar lipid analysis revealed that phosphatidylethanolamine (PE) was commonly present in the two novel strains. Additionally, strain KFT8ᵀ contained one unidentified aminophospholipid (APL), three unidentified phospholipids (PLs), and three unidentified lipids (Ls), whereas strain CG01ᵀ contained two APLs, two PLs, and three Ls (Fig. S4).

### Taxonomic conclusion

In this study, the two novel strains KFT8^T^ and CG01^T^ were phylogenetically related to the genus *Bacteroides* but exhibited distinct features compared to other members of the genus based on polyphasic characterizations. While both strains shared several typical features of the genus *Bacteroides*, such as bile tolerance, non-motility, non-spore formation, and the presence of genes encoding putative carbohydrate-active enzymes associated with starch utilization, minor differences were identified in their biochemical and chemotaxonomic profiles. In particular, strain KFT8ᵀ and *B. facilis* KCTC 25155^T^ both exhibited C_18:1_ *ω*9*c* as the predominant fatty acid, whereas *B. ovatus* KCTC 5827^T^ and *B. xylanisolvens* KCTC 15192^T^ showed *anteiso*-C_15:0_ as the most abundant component. In contrast, strain CG01ᵀ exhibited summed feature 11 (comprising *iso*-C_17:0_ 3OH and/or C_18:2_ DMA) as its major fatty acid, while *B. nordii* KCTC 25023^T^ and *B. salyersiae* KCTC 5799^T^ showed a predominance of anteiso-C_15:0_.

Genome-based analyses, including OrthoANI and dDDH values, yielded results below the accepted threshold for species delineation, indicating that strains KFT8^T^ and CG01^T^ are distinguishable from their closely related species within the genus *Bacteroides*. These findings support the classification of both strains as novel species of the genus *Bacteroides*, for which the names *Bacteroides celer* sp. nov. for strain KFT8^T^ and *Bacteroides mucinivorans* sp. nov. for strain CG01^T^ are proposed.

In addition, comparative analyses based on 16S rRNA gene and whole-genome sequences revealed that the type strains of *B. koreensis* and *B. kribbi* are genetically identical and indistinguishable from *B. ovatus* at the species level. Therefore, we propose that *B. koreensis* Shin et al. 2017 and *B. kribbi* Shin et al. 2017 be reclassified as later heterotypic synonyms of *B. ovatus* Eggerth and Gagnon 1933 (Approved Lists 1980). 

### Description of *Bacteroides celer* sp. nov.

*Bacteroides celer* (ce'ler. L. masc. adj. *celer*, rapid, pertaining to the fast growth of the species).

Cells are Gram-stain-negative, strictly anaerobic, bile-tolerant, non-motile, non-spore-forming, and rod-shaped, measuring 2–3 µm in length and 1.0–1.5 µm in width. Colonies are circular, cream-colored, translucent, moist, and glistening, with entire margins on GAM agar under optimal growth conditions after 3 days of incubation. Oxidase and catalase activities are negative. Growth occurs at 25–37°C (optimum, 37°C), in 0–5% (w/v) NaCl (optimum, 1%), and at pH 6–9 (optimum, pH 8). Positive for the following reactions: acidification of D-glucose, D-mannitol, D-lactose, D-saccharose, D-maltose, salicin, D-xylose, L-arabinose, glycerol, D-cellobiose, D-mannose, D-melezitose, D-raffinose, D-sorbitol, D-rhamnose, D-trehalose, and hydrolysis of esculin ferric citrate (*β*-glucosidase) (API 20A); arginine dihydrolase, *α*-galactosidase, *β*-galactosidase, *α*-glucosidase, *β*-glucosidase, *α*-arabinosidase, *N*-acetyl-*β*-glucosaminidase, *α*-fucosidase, alkaline phosphatase, mannose, raffinose, glutamic acid decarboxylase, and arylamidase activities for leucyl glycine, alanine, and glutamyl-glutamic acid (API Rapid ID 32A); and fermentation of L-arabinose, D-xylose, D-galactose, D-glucose, D-fructose, D-mannose, L-rhamnose, D-mannitol, *N*-acetylglucosamine, amygdalin, esculin, D-cellobiose, D-maltose, D-lactose, D-melibiose, sucrose, D-trehalose, inulin, D-melezitose, D-raffinose, starch, glycogen, gentiobiose, D-turanose, and L-fucose (API 50CH). Negative for the following reactions: urease, *β*-galactosidase-6-phosphate, *β*-glucuronidase, glutamic acid decarboxylase, reduction of nitrates to nitrites, indole production from L-tryptophan, and arylamidase activities for arginine, proline, phenylalanine, leucine, pyroglutamic acid, tyrosine, glycine, histidine, and serine (API Rapid ID 32A); indole formation, urease, and gelatin hydrolysis (protease) (API 20A); and fermentation of glycerol, erythritol, D-arabinose, D-ribose, L-xylose, D-adonitol, methyl-*β*-D-xyloside, L-sorbose, dulcitol, inositol, D-sorbitol, methyl-*α*-D-mannoside, methyl-*α*-D-glucoside, arbutin, salicin, xylitol, D-lyxose, D-tagatose, D-fucose, D-arabitol, L-arabitol, gluconate, 2-keto-gluconate, and 5-keto-gluconate (API 50CH). The major cellular fatty acids include C_18:1_ *ω*9*c*, C_16:0_ 3OH, and *iso*-C_14:0_. MK-11 is the sole respiratory quinone. The polar lipid profile consists of phosphatidylethanolamine, one unidentified aminophospholipid, three unidentified phospholipids, and three unidentified lipids. The metabolic end-products are succinic acid, acetic acid, and lactic acid. The genomic DNA G + C content of the type strain is 41.8%.

The type strain is KFT8^T^ (= KCTC 15614^T^ = JCM 30116^T^), which was isolated from the feces of a 23-year-old healthy male. The GenBank/EMBL/DDBJ accession numbers for the 16S rRNA gene and whole-genome sequences of strain KFT8^T^ are KY703634 and CP178209, respectively.

### Description of *Bacteroides mucinivorans* sp. nov.

*Bacteroides mucinivorans* (mu.ci.ni.vo’rans. N.L. neut. n. *mucinum*, mucin; L. pres. part. *vorans*, eating; N.L. part. adj. *mucinivorans*, mucin-eating).

Cells are Gram-stain-negative, obligate anaerobes, bile-tolerant, non-motile, non-spore-forming, and rod-shaped, measuring 2.0–3.5 µm in length and 1.0 µm in width. Colonies are circular, cream-colored, translucent, moist, and glistening, with entire margins on GAM agar under optimal growth conditions at 37°C after 3 days of incubation. Oxidase and catalase activities are negative. Growth occurs at 25–37°C (optimum, 37°C), in 0–5% (w/v) NaCl (optimum, 1%), and at pH 6–9 (optimum, pH 8). Positive for the following reactions: acidification of D-glucose, D-lactose, D-saccharose, D-maltose, D-xylose, D-cellobiose, D-mannose, D-raffinose, D-rhamnose, and hydrolysis of esculin ferric citrate (*β*-glucosidase) (API 20A); arginine dihydrolase, *α*-glucosidase, *β*-glucosidase, *N*-acetyl-*β*-glucosaminidase, glutamic acid decarboxylase, alkaline phosphatase, and arylamidase activities for leucyl glycine and alanine (API Rapid ID 32A); and fermentation of D-xylose, D-galactose, D-glucose, D-fructose, D-mannose, L-rhamnose, *N*-acetylglucosamine, amygdalin, esculin, D-cellobiose, D-maltose, sucrose, D-lactose, inulin, D-raffinose, starch, glycogen, and gentiobiose (API 50CH). Negative for the following reactions: urease, *α*-galactosidase, *β*-galactosidase, *β*-galactosidase-6-phosphate, *α*-arabinosidase, *β*-glucuronidase, mannose, raffinose, *α*-fucosidase, reduction of nitrates to nitrites, indole production from L-tryptophan, and arylamidase activities for arginine, proline, phenylalanine, leucine, pyroglutamic acid, tyrosine, glycine, histidine, glutamyl-glutamic acid, and serine (API Rapid ID 32A); indole formation from L-tryptophan, urease, D-mannitol, salicin, L-arabinose, glycerol, D-melezitose, D-sorbitol, D-trehalose, and gelatin hydrolysis (protease) (API 20A); and fermentation of glycerol, erythritol, D-arabinose, L-arabinose, D-ribose, L-xylose, D-adonitol, methyl-*β*-D-xyloside, L-sorbose, dulcitol, inositol, D-sorbitol, D-mannitol, methyl-*α*-D-mannoside, methyl-*α*-D-glucoside, arbutin, salicin, D-melibiose, D-trehalose, D-melezitose, xylitol, D-turanose, D-lyxose, D-tagatose, D-fucose, L-fucose, D-arabitol, L-arabitol, 2-keto-gluconate, gluconate, and 5-keto-gluconate (API 50CH). The major cellular fatty acids are summed feature 11 (comprising *iso*-C_17:0_ 3OH and/or C_18:2_ DMA), *anteiso*-C_15:0_, and C_18:1_ *ω*9*c*. MK-11 is the sole respiratory quinone. The polar lipid profile consists of phosphatidylethanolamine, two unidentified aminophospholipids, two unidentified phospholipids, and three unidentified lipids. The metabolic end-products are succinic acid, acetic acid, and formic acid. The genomic DNA G + C content of the type strain is 40.3%.

The type strain is CG01^T^ (= KCTC 15613^T^ = JCM 36010^T^), which was isolated from the feces of a 28-year-old healthy female. The GenBank/EMBL/DDBJ accession numbers for the 16S rRNA gene and whole-genome sequences of strain CG01^T^ are OR272335 and CP178179, respectively.

## Figures and Tables

**Table 1.** Differential characteristics of the two novel strains compared to phylogenetically related species. All strains were positive for the acidification of D-glucose, D-lactose, D-saccharose, D-maltose, D-xylose, hydrolysis of esculin ferric citrate (*β*-glucosidase), D-mannose, D-rhamnose (API 20A), arginine dihydrolase, *β*-glucosidase, *N*-acetyl-*β*-glucosaminidase, alkaline phosphatase, and arylamidase activities for leucyl glycine and alanine (API Rapid ID 32A). In the API 50 CH test strip, all strains tested positive for D-xylose, D-galactose, D-glucose, D-fructose, D-mannose, amygdalin, esculin, D-maltose, D-lactose, sucrose, inulin, D-raffinose, starch, glycogen, and gentiobiose. All data were obtained in this study. +, Positive; –, negative; w, weakly positive; ND, not determined.

Characteristics	KFT8^T^	*B. facilis* KCTC 25155^T^	*B. ovatus* KCTC 5827^T^	*B. xylanisolvens* KCTC 15192^T^	CG01^T^	*B. nordii* KCTC 25023^T^	*B. salyersiae* KCTC 5799^T^
Temperature range (optimum) (°C)	25–37 (37)	(37)^a^	15–40^b^	15–40^b^	20–42	ND	ND
pH range (optimum)	6–9 (8)	(7.0–7.5)^a^	5.5–10^b^	5.5–10^b^	6–9 (8)	ND	ND
NaCl range (optimum) (%, w/v)	0–5 (1)	ND	1–4^b^	1–6^b^	0–5 (1)	ND	ND
Enzyme activities (API Rapid ID 32A)							
*α*-Galactosidase, *β*-galactosidase	+	+	+	+	–	–	–
*α*-Glucosidase	+	+	+	+	+	+	–
*α*-Arabinosidase, mannose, raffinose	w	–	w	w	–	–	–
Glutamic acid decarboxylase	–	+	+	+	+	+	+
Indole production (from L-tryptophan)	–	–	+	w	–	–	–
Glutamic acid decarboxylase	w	+	w	+	–	+	+
Acid production (API 20A)							
D-Mannitol, salicin, D-melezitose, D-sorbitol, D-trehalose	+	+	+	w	–	–	–
Arabinose	+	+	+	+	–	–	+
Glycerol	w	–	w	w	–	–	–
Cellobiose	+	+	+	+	w	–	–
Raffinose	+	+	+	+	w	–	w
Acid production (API 50CH)							
D-Arabinose, arbutin, xylitol	–	+	–	–	–	–	–
D-Ribose	–	–	+	+	–	–	–
L-Rhamnose, *N*-acetylglucosamine	+	–	+	+	+	+	w
D-Mannitol	w	–	+	–	–	–	–
D-Cellobiose	w	+	+	+	+	–	w
D-Melibiose	+	+	+	+	–	–	–
D-Trehalose, D-melezitose, D-turanose	w	–	+	w	–	–	–
L-Fucose	w	–	+	+	–	+	+

Part of the data for growth range and optimum conditions were obtained from Liu et al. (2021)^a^ and Shin et al. (2017)^b^.

**Table 2.** Cellular fatty acid compositions (%) of the two novel strains compared with phylogenetically related species. Strains: 1, KFT8^T^; 2, *B. facilis* KCTC 25155^T^; 3, *B. ovatus* KCTC 5827^T^; 4, *B. xylanisolvens* KCTC 15192^T^; 5, CG01^T^; 6, *B. nordii* KCTC 25023^T^; 7, *B. salyersiae* KCTC 5799^T^ –, Not detected; tr, trace (< 0.5%). The major fatty acids (> 10%) are highlighted in bold. All data were obtained in this study.

Cellular fatty acid (%)	1	2	3	4	5	6	7
Saturated straight chain							
C_14:0_	5.0	3.0	3.8	1.2	0.7	0.7	0.5
C_15:0_	3.0	**12.8**	1.9	0.9	0.6	0.8	0.5
C_16:0_	9.4	7.7	5.7	4.9	4.3	3.6	3.0
C_18:0_	5.2	3.0	tr	1.2	tr	0.6	tr
Hydroxy acids							
C_15:0_ 3OH	1.1	2.5	0.7	tr	tr	tr	–
C_16:0_ 3OH	**12.2**	**12.7**	**13.2**	8.0	6.2	4.7	6.0
C_17:0_ 3OH	tr	2.1	tr	tr	tr	tr	–
Branched acids							
*iso*-C_14:0_	**11.0**	9.9	0.9	0.6	tr	0.7	0.7
*iso*-C_15:0_	2.6	3.6	**11.1**	9.4	**12.5**	**15.7**	**15.8**
*iso*-C_16:0_	2.3	1.4	tr	0.6	0.8	0.8	tr
*iso*-C_17:0_	–	–	–	0.6	2.1	0.7	tr
*anteiso*-C_15:0_	**10.2**	**10.0**	**30.6**	**28.8**	**23.3**	**25.9**	**27.3**
*anteiso*-C_17:0_	tr	tr	tr	1.5	1.2	0.5	tr
*anteiso*-C_17:0_ 3OH	0.9	tr	3.1	3.7	2.7	2.3	2.1
Unsaturated straight chain							
C_18:1_ *ω*9*c*	**21.0**	**17.8**	**10.2**	**14.4**	**13.7**	**13.1**	**14.6**
C_18:2_ *ω*6,9*c*	0.9	0.7	tr	0.6	0.6	0.5	0.6
Summed features^*^							
3	–	–	tr	tr	tr	tr	1.1
9	9.0	5.7	1.5	1.8	1.3	1.3	0.9
10	2.3	1.6	0.6	1.1	0.7	0.7	0.6
11	1.5	2.7	**14.1**	**18.8**	**27.2**	**25.8**	**23.6**
13	tr	–	–	–	–	–	0.6

* Summed feature 3 contained *iso*-C_15:0_ aldehyde. Summed feature 9 contained *iso*-C_16:0_ 3OH. Summed feature 10 contained C_18:1_ *ω7c*. Summed feature 11 contained *iso*-C_17:0_ 3OH and/or C_18:2_ DMA. Summed feature 13 contained *anteiso*-C_15:0_ DMA.

## Data Availability

All data generated in this study are included in the article and its supplementary information. The GenBank data generated in this study are available from NCBI database (https://www.ncbi.nlm.nih.gov/).

## Supplementary Information

Table S1. General genomic features of the two novel strains and closely related type strains within the genus *Bacteroides*. Strains: 1, KFT8^T^; 2, *B. facilis* NSJ-77^T^; 3, *B. ovatus* ATCC 8483^T^; 4, *B. xylanisolvens* XB1A^T^; 5, CG01^T^; 6, *B. nordii* JCM 12987^T^; 7, *B. salyersiae* DSM 18765^T^; ND, not determined

Table S2. The pairwise comparison table of orthologous average nucleotide identity (OrthoANI; lower left), digital DNA-DNA hybridization (dDDH; upper right) values (%) between the two novel strains and the closely related type strains within the genus *Bacteroides*. Strains: 1, KFT8^T^; 2, *B. facilis* NSJ-77^T^; 3, *B. ovatus* ATCC 8483^T^; 4, *B. xylanisolvens* XB1A^T^; 5, CG01^T^; 6, *B. nordii* JCM 12987^T^; 7, *B. salyersiae* DSM 18765^T^

Table S3. The relative abundance (%) of CDSs within subsystem categories of the two novel strains and closely related type strains in the genus *Bacteroides* as annotated using RAST. Strains: 1, KFT8^T^; 2, *B. facilis* NSJ-77^T^; 3, *B. ovatus* ATCC 8483^T^; 4, *B. xylanisolvens* XB1A^T^; 5, CG01^T^; 6, *B. nordii* JCM 12987^T^; 7, *B. salyersiae* DSM 18765^T^

Table S4. The relative abundance (%) of annotated functional family domains of carbohydrate-active enzymes (CAZymes) for the two novel strains and closely related type strains in the genus *Bacteroides*. Strains: 1, KFT8^T^; 2, *B. facilis* NSJ-77^T^; 3, *B. ovatus* ATCC 8483^T^; 4, *B. xylanisolvens* XB1A^T^; 5, CG01^T^; 6, *B. nordii* JCM 12987^T^; 7, *B. salyersiae* DSM 18765^T^

Table S5. The pairwise comparison table of orthologous average nucleotide identity (OrthoANI; lower left) and digital DNA-DNA hybridization (dDDH; upper right) values (%) among *Bacteroides ovatus* ATCC 8483^T^, *B. koreensis* JCM 31393^T^, and *B. kribbi* JCM 31391^T^. Strains: 1, *B. ovatus* ATCC 8483^T^; 2, *B. koreensis* JCM 31393^T^; 3, *B. kribbi* JCM 31391^T^

Fig. S1. Phylogenetic tree was constructed based on 16S rRNA gene sequences using NJ algorithms with the addition of *B. koreensis* KCTC 15520^T^ and *B. kribbi* KCTC 15460^T^. Bootstrap values over 70% based on NJ/ML/MP algorithms are shown at nodes as a percentage of 1,000 replicates. Filled circles represent branches consistently recovered in all three algorithms. Scale bar indicates 0.02 substitutions per nucleotide.

Fig. S2. Phylogenomic position of *B. koreensis* JCM 31393^T^ and *B. kribbi* JCM 31391^T^ was constructed using UBCG pipeline. The numbers at nodes are bootstrap values. Scale bar indicates 0.05 substitutions per nucleotide.

Fig. S3. Transmission electron microscopy of strains (A and B) KFT8^T^ and (C and D) CG01^T^. Cells were grown on the GAM agar at 37℃ for 3 days.

Fig. S4. Two-dimensional thin-layer chromatograms on the silica plates of total lipids of strains (A) KFT8^T^ and (B) CG01^T^. Components were detected by spraying reagents with 5% ethanolic molybdatophosphoric acid. PE, phosphatidylethanolamine; PL, unidentified phospholipid; APL, unidentified aminophospholipid; L, unidentified lipid.
