# Erratum: *Bacteroides celer* sp. nov. and *Bacteroides mucinivorans* sp. nov., isolated from human feces, and the reclassification of *Bacteroides koreensis* Shin et al. 2017 and *Bacteroides kribbi* Shin et al. 2017 as later heterotypic synonyms of *Bacteroides ovatus* Eggerth and Gagnon 1933 (Approved Lists 1980)

**DOI:** 10.71150/jm.2507100

**Published:** 2025-07-21

**Authors:** Ah-In Yang, Bora Kim, Woorim Kang, Hae-In Joe, Na-Ri Shin

**Affiliations:** 1 Biological Resource Center, Korea Research Institute of Bioscience and Biotechnology, Jeongeup 56212, Republic of Korea; 2 Department of Biology, Kyung Hee University, Seoul 02447, Republic of Korea; 3 Department of Biological Science, Kangwon National University, Chuncheon 24341, Republic of Korea

**Erratum: Journal of Microbiogy. 2025. 63: e2502006.**

https://doi.org/10.71150/jm.2502006

The original online version of this article was revised:

This article contains two errors: one in the Results and Discussion section and the other in Table 1.

## Results and Discussion

### Description of *Bacteroides celer* sp. nov.

­

**The originally published version read as follows:**

The type strain is KFT8^T^ (= KCTC 15614^T^ = JCM 30116^T^), which was isolated from the feces of a 23-year-old healthy male.

­

**It should be read as follows:**

The type strain is KFT8^T^ (= KCTC 15614^T^ = JCM 36011^T^), which was isolated from the feces of a 23-year-old healthy male.

## Published Table 1

The value for “Temperature range (optimum) (°C)” of strain CG01^T^ was published incorrectly.[Table t1-jm-2507100]

**Table 1. t1-jm-2507100:** Differential characteristics of the two novel strains compared to phylogenetically related species. All strains were positive for the acidification of D-glucose, D-lactose, D-saccharose, D-maltose, D-xylose, hydrolysis of esculin ferric citrate (*β*-glucosidase), D-mannose, D-rhamnose (API 20A), arginine dihydrolase, *β*-glucosidase, *N*-acetyl-*β*-glucosaminidase, alkaline phosphatase, and arylamidase activities for leucyl glycine and alanine (API Rapid ID 32A). In the API 50 CH test strip, all strains tested positive for D-xylose, D-galactose, D-glucose, D-fructose, D-mannose, amygdalin, esculin, D-maltose, D-lactose, sucrose, inulin, D-raffinose, starch, glycogen, and gentiobiose. All data were obtained in this study. +, Positive; –, negative; w, weakly positive; ND, not determined.

Characteristics	KFT8^T^	*B. facilis* KCTC 25155^T^	*B. ovatus* KCTC 5827^T^	*B. xylanisolvens* KCTC 15192^T^	CG01^T^	*B. nordii* KCTC 25023^T^	*B. salyersiae* KCTC 5799^T^
Temperature range (optimum) (°C)	25–37 (37)	(37)^a^	15–40^b^	15–40^b^	20–42	ND	ND
pH range (optimum)	6–9 (8)	(7.0–7.5)^a^	5.5–10^b^	5.5–10^b^	6–9 (8)	ND	ND
NaCl range (optimum) (%, w/v)	0–5 (1)	ND	1–4^b^	1–6^b^	0–5 (1)	ND	ND
Enzyme activities (API Rapid ID 32A)							
*α*-Galactosidase, *β*-galactosidase	+	+	+	+	–	–	–
*α*-Glucosidase	+	+	+	+	+	+	–
*α*-Arabinosidase, mannose, raffinose	w	–	w	w	–	–	–
Glutamic acid decarboxylase	–	+	+	+	+	+	+
Indole production (from L-tryptophan)	–	–	+	w	–	–	–
Glutamic acid decarboxylase	w	+	w	+	–	+	+
Acid production (API 20A)							
D-Mannitol, salicin, D-melezitose, D-sorbitol, D-trehalose	+	+	+	w	–	–	–
Arabinose	+	+	+	+	–	–	+
Glycerol	w	–	w	w	–	–	–
Cellobiose	+	+	+	+	w	–	–
Raffinose	+	+	+	+	w	–	w
Acid production (API 50CH)							
D-Arabinose, arbutin, xylitol	–	+	–	–	–	–	–
D-Ribose	–	–	+	+	–	–	–
L-Rhamnose, *N*-acetylglucosamine	+	–	+	+	+	+	w
D-Mannitol	w	–	+	–	–	–	–
D-Cellobiose	w	+	+	+	+	–	w
D-Melibiose	+	+	+	+	–	–	–
D-Trehalose, D-melezitose, D-turanose	w	–	+	w	–	–	–
L-Fucose	w	–	+	+	–	+	+

Part of the data for growth range and optimum conditions were obtained from Liu et al. (2021)^a^ and Shin et al. (2017)^b^.

## Corrected Table 1

The value for “Temperature range (optimum) (°C)” of strain CG01^T^ has been corrected.[Table t2-jm-2507100]

**Table 1. t2-jm-2507100:** Differential characteristics of the two novel strains compared to phylogenetically related species. All strains were positive for the acidification of D-glucose, D-lactose, D-saccharose, D-maltose, D-xylose, hydrolysis of esculin ferric citrate (*β*-glucosidase), D-mannose, D-rhamnose (API 20A), arginine dihydrolase, *β*-glucosidase, *N*-acetyl-*β*-glucosaminidase, alkaline phosphatase, and arylamidase activities for leucyl glycine and alanine (API Rapid ID 32A). In the API 50 CH test strip, all strains tested positive for D-xylose, D-galactose, D-glucose, D-fructose, D-mannose, amygdalin, esculin, D-maltose, D-lactose, sucrose, inulin, D-raffinose, starch, glycogen, and gentiobiose. All data were obtained in this study. +, Positive; –, negative; w, weakly positive; ND, not determined.

Characteristics	KFT8^T^	*B. facilis* KCTC 25155^T^	*B. ovatus* KCTC 5827^T^	*B. xylanisolvens* KCTC 15192^T^	CG01^T^	*B. nordii* KCTC 25023^T^	*B. salyersiae* KCTC 5799^T^
Temperature range (optimum) (°C)	25–37 (37)	(37)^a^	15–40^b^	15–40^b^	20–42 (37)	ND	ND
pH range (optimum)	6–9 (8)	(7.0–7.5)^a^	5.5–10^b^	5.5–10^b^	6–9 (8)	ND	ND
NaCl range (optimum) (%, w/v)	0–5 (1)	ND	1–4^b^	1–6^b^	0–5 (1)	ND	ND
Enzyme activities (API Rapid ID 32A)							
*α*-Galactosidase, *β*-galactosidase	+	+	+	+	–	–	–
*α*-Glucosidase	+	+	+	+	+	+	–
*α*-Arabinosidase, mannose, raffinose	w	–	w	w	–	–	–
Glutamic acid decarboxylase	–	+	+	+	+	+	+
Indole production (from L-tryptophan)	–	–	+	w	–	–	–
Glutamic acid decarboxylase	w	+	w	+	–	+	+
Acid production (API 20A)							
D-Mannitol, salicin, D-melezitose, D-sorbitol, D-trehalose	+	+	+	w	–	–	–
Arabinose	+	+	+	+	–	–	+
Glycerol	w	–	w	w	–	–	–
Cellobiose	+	+	+	+	w	–	–
Raffinose	+	+	+	+	w	–	w
Acid production (API 50CH)							
D-Arabinose, arbutin, xylitol	–	+	–	–	–	–	–
D-Ribose	–	–	+	+	–	–	–
L-Rhamnose, *N*-acetylglucosamine	+	–	+	+	+	+	w
D-Mannitol	w	–	+	–	–	–	–
D-Cellobiose	w	+	+	+	+	–	w
D-Melibiose	+	+	+	+	–	–	–
D-Trehalose, D-melezitose, D-turanose	w	–	+	w	–	–	–
L-Fucose	w	–	+	+	–	+	+

Part of the data for growth range and optimum conditions were obtained from Liu et al. (2021)^a^ and Shin et al. (2017)^b^.

