# Supplementary material for: Bacteroides celer sp. nov. and Bacteroides mucinivorans sp. nov., isolated from human feces, and the reclassification of Bacteroides koreensis Shin et al. 2017 and Bacteroides kribbi Shin et al. 2017 as later heterotypic synonyms of Bacteroides ovatus Eggerth and Gagnon 1933 (Approved Lists 1980)
Source: J Microbiol. 2025 Jun 30;63(6):e2502006. doi: 10.71150/jm.2502006 (PMC13577008; doi:10.71150/jm.2502006)
Supplement: Supplementary file 1 [file jm-2502006-Supplementary-Fig-S1.pdf]

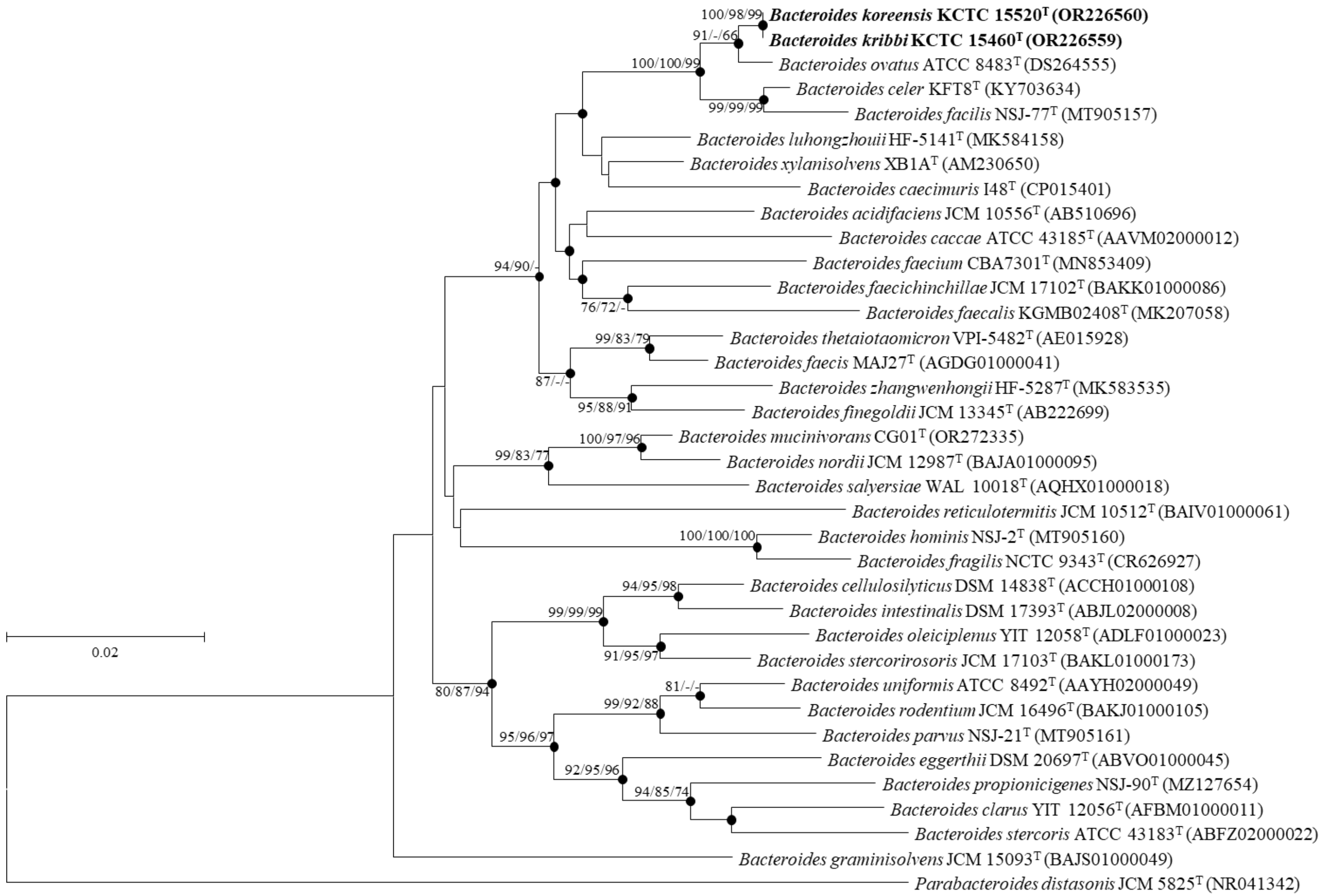

Fig. S1. Phylogenetic tree was constructed based on 16S rRNA gene sequences using NJ algorithms with the addition of *B. koreensis* KCTC 15520^T and *B. kribbi* KCTC 15460^T. Bootstrap values over 70% based on NJ/ML/MP algorithms are shown at nodes as a percentage of 1,000 replicates. Filled circles represent branches consistently recovered in all three algorithms. Scale bar indicates 0.02 substitutions per nucleotide.
