# Supplementary material for: Bacteroides celer sp. nov. and Bacteroides mucinivorans sp. nov., isolated from human feces, and the reclassification of Bacteroides koreensis Shin et al. 2017 and Bacteroides kribbi Shin et al. 2017 as later heterotypic synonyms of Bacteroides ovatus Eggerth and Gagnon 1933 (Approved Lists 1980)
Source: J Microbiol. 2025 Jun 30;63(6):e2502006. doi: 10.71150/jm.2502006 (PMC13577008; doi:10.71150/jm.2502006)
Supplement: Supplementary file 4 [file jm-2502006-Supplementary-Fig-S4.pdf]

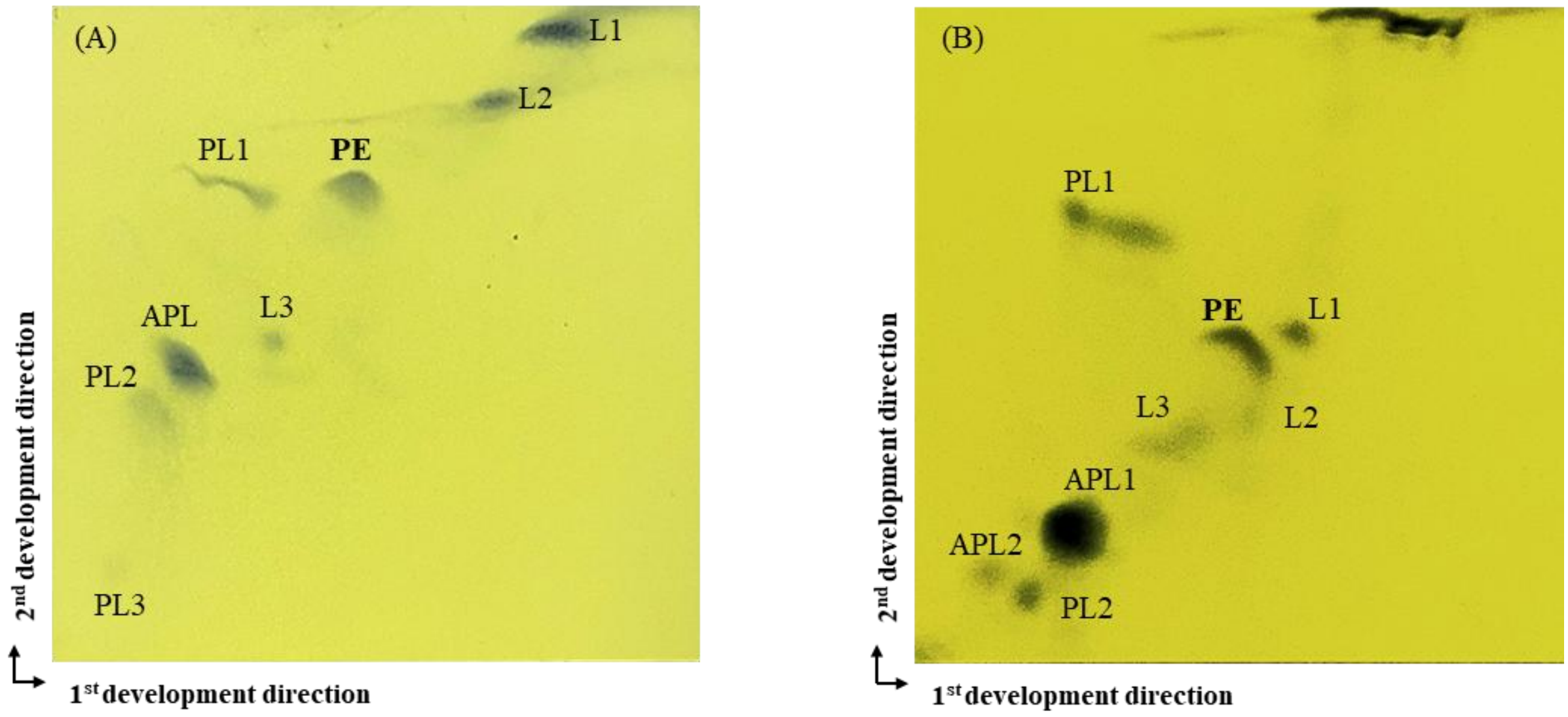

Fig. S4. Two-dimensional thin-layer chromatograms on the silica plates of total lipids of strains (A) KFT8^T and (B) CG01^T. Components were detected by spraying reagents with 5% ethanolic molybdatophosphoric acid.

PE, phosphatidylethanolamine; PL, unidentified phospholipid; APL, unidentified aminophospholipid; L, unidentified lipid.
