# Supplementary material for: Bacteroides celer sp. nov. and Bacteroides mucinivorans sp. nov., isolated from human feces, and the reclassification of Bacteroides koreensis Shin et al. 2017 and Bacteroides kribbi Shin et al. 2017 as later heterotypic synonyms of Bacteroides ovatus Eggerth and Gagnon 1933 (Approved Lists 1980)
Source: J Microbiol. 2025 Jun 30;63(6):e2502006. doi: 10.71150/jm.2502006 (PMC13577008; doi:10.71150/jm.2502006)
Supplement: Supplementary file 5 [file jm-2502006-Supplementary-Table-S1.pdf]

Table S1. General genomic features of the two novel strains and closely related type strains within the genus *Bacteroides*.

Strains: 1, KFT8^T; 2, *B. facilis* NSJ-77^T; 3, *B. ovatus* ATCC 8483^T; 4, *B. xylanisolvens* XB1A^T; 5, CG01^T; 6, *B. nordii* JCM 12987^T; 7, *B. salyersiae* DSM 18765^T; ND, not determined

**Features**	**1**	**2**	**3**	**4**	**5**	**6**	**7**
**Size (bp)**	6,031,385	6,746,034	6,472,489	5,976,145	6,053,331	5,591,555	5,315,450
**Contigs**	single, complete	single, complete	single, complete	230	single, complete	118	6
**G + C content (%)**	41.8	41.7	41.9	41.9	40.3	40.6	41.9
**Number of CDS**	4,910	5,244	5,081	5,149	4,727	5,340	4,389
**Number of rRNA (5S/16S/23S)**	15 (5/5/5)	15 (5/5/5)	15 (5/5/5)	ND	15 (5/5/5)	3 (1/1/1)	14 (4/4/6)
**Number of tRNA**	24	24	23	ND	23	23	21
**GenBank Accession**	CP178209	CP060488	CP012938	FP929033	CP178179	BAJA00000000	AQHX00000000
