# Supplementary material for: Bacteroides celer sp. nov. and Bacteroides mucinivorans sp. nov., isolated from human feces, and the reclassification of Bacteroides koreensis Shin et al. 2017 and Bacteroides kribbi Shin et al. 2017 as later heterotypic synonyms of Bacteroides ovatus Eggerth and Gagnon 1933 (Approved Lists 1980)
Source: J Microbiol. 2025 Jun 30;63(6):e2502006. doi: 10.71150/jm.2502006 (PMC13577008; doi:10.71150/jm.2502006)
Supplement: Supplementary file 7 [file jm-2502006-Supplementary-Table-S3.pdf]

Table S3. The relative abundance (%) of CDSs within subsystem categories of the two novel strains and closely related type strains in the genus *Bacteroides* as annotated using RAST. Strains: 1, KFT8^{T}; 2, *B. facilis* NSJ-77^{T}; 3, *B. ovatus* ATCC 8483^{T}; 4, *B. xylanisolvens* XB1A^{T}; 5, CG01^{T}; 6, *B. nordii* JCM 12987^{T}; 7, *B. salyersiae* DSM 18765^{T}

**Subsystem Category (%)**	**1**	**2**	**3**	**4**	**5**	**6**	**7**
Carbohydrates	18.9	18.6	19.5	19.0	18.4	18.6	17.4
Amino acids and derivatives	15.2	13.8	14.6	14.0	14.8	14.0	14.9
Protein metabolism	14.5	13.8	13.9	11.1	15.8	13.6	14.9
Cofactors, vitamins, prosthetic groups, pigments	10.4	10.1	10.2	9.9	9.2	9.2	9.8
DNA metabolism	6.4	5.4	6.1	6.0	5.4	5.1	4.9
Nucleosides and nucleotides	6.1	6.0	6.0	5.9	5.8	6.1	6.0
Respiration	5.4	4.9	5.0	4.9	5.0	4.4	4.9
RNA metabolism	3.8	2.9	2.9	2.7	2.6	2.6	2.6
Virulence, disease, and defense	2.6	2.8	2.5	2.6	2.4	3.4	2.9
Membrane transport	2.5	2.3	2.5	2.3	2.6	3.1	2.6
Phosphorus metabolism	2.3	2.2	2.4	1.9	1.1	1.2	1.7
Fatty acids, lipids, and isoprenoids	2.2	2.0	2.1	1.9	1.8	2.0	1.5
Cell wall and capsule	2.1	3.6	2.2	3.5	4.2	4.1	2.9
Sulfur metabolism	2.1	2.6	1.8	1.5	1.3	1.9	1.8
Stress response	1.4	1.2	1.3	1.2	2.0	1.7	2.0
Potassium metabolism	0.8	0.8	0.8	0.9	0.7	0.7	0.7
Phages, prophages, transposable elements, plasmids	0.8	4.2	3.3	7.9	4.3	5.1	5.0
Nitrogen metabolism	0.7	0.8	0.7	0.9	0.8	0.9	0.6
Regulation and cell signaling	0.5	0.5	0.7	0.6	0.5	0.7	0.5
Miscellaneous	0.3	0.3	0.3	0.2	0.2	0.2	0.2
Secondary metabolism	0.3	0.3	0.3	0.3	0.3	0.3	0.3
Cell division and cell cycle	0.2	0.2	0.2	0.2	0.3	0.4	1.5
Metabolism of aromatic compounds	0.2	0.2	0.2	0.2	0.2	0.2	0.2
Dormancy and sporulation	0.2	0.2	0.2	0.2	0.2	0.1	0.2
Iron acquisition and metabolism	0.1	0.1	0.2	0.1	0.1	0.3	0.0
Total	100	100	100	100	100	100	100
