# Supplementary material for: Bacteroides celer sp. nov. and Bacteroides mucinivorans sp. nov., isolated from human feces, and the reclassification of Bacteroides koreensis Shin et al. 2017 and Bacteroides kribbi Shin et al. 2017 as later heterotypic synonyms of Bacteroides ovatus Eggerth and Gagnon 1933 (Approved Lists 1980)
Source: J Microbiol. 2025 Jun 30;63(6):e2502006. doi: 10.71150/jm.2502006 (PMC13577008; doi:10.71150/jm.2502006)
Supplement: Supplementary file 8 [file jm-2502006-Supplementary-Table-S4.pdf]

Table S4. The relative abundance (%) of annotated functional family domains of carbohydrate-active enzymes (CAZymes) for the two novel strains and closely related type strains in the genus *Bacteroides*.

Strains: 1, KFT8^T; 2, *B. facilis* NSJ-77^T; 3, *B. ovatus* ATCC 8483^T; 4, *B. xylanisolvens* XB1A^T; 5, CG01^T; 6, *B. nordii* JCM 12987^T; 7, *B. salyersiae* DSM 18765^T

**Family domains (%)**	**1**	**2**	**3**	**4**	**5**	**6**	**7**
Glycoside hydrolase (GH)	60.2	63.7	64.2	59.2	60.5	56.5	56.4
Glycosyl transferase (GT)	16.6	12.4	12.7	21.2	16.9	22.3	21.8
Carbohydrate-binding module (CBM)	9.4	8.1	8.9	6.8	7.2	6.6	9.2
Carbohydrate esterase (CE)	7.6	8.0	7.6	7.5	6.1	6.6	6.7
Polysaccharide lyase (PL)	6.2	7.8	6.6	5.3	9.3	7.9	5.9
Total	100	100	100	100	100	100	100
