# Supplementary material for: Bacteroides celer sp. nov. and Bacteroides mucinivorans sp. nov., isolated from human feces, and the reclassification of Bacteroides koreensis Shin et al. 2017 and Bacteroides kribbi Shin et al. 2017 as later heterotypic synonyms of Bacteroides ovatus Eggerth and Gagnon 1933 (Approved Lists 1980)
Source: J Microbiol. 2025 Jun 30;63(6):e2502006. doi: 10.71150/jm.2502006 (PMC13577008; doi:10.71150/jm.2502006)
Supplement: Supplementary file 9 [file jm-2502006-Supplementary-Table-S5.pdf]

Table S5. The pairwise comparison table of orthologous average nucleotide identity (OrthoANI; lower left) and digital DNA-DNA hybridization (dDDH; upper right) values (%) among *Bacteroides ovatus* ATCC 8483^T, *B. koreensis* JCM 31393^T, and *B. kribbi* JCM 31391^T. Strains: 1, *B. ovatus* ATCC 8483^T; 2, *B. koreensis* JCM 31393^T; 3, *B. kribbi* JCM 31391^T

	**1**	**2**	**3**
**1**	–	87.8	87.9
**2**	98.5	–	100
**3**	98.5	99.9	–
